# Temporal and Spatial Expression of CCN Genes in Zebrafish

**DOI:** 10.1002/dvdy.22279

**Published:** 2010-04-09

**Authors:** Carol A Fernando, Patricia A Conrad, Cynthia F Bartels, Tomas Marques, Michael To, Stephanie A Balow, Yukio Nakamura, Matthew L Warman

**Affiliations:** 1 Department of Genetics, Case Western Reserve UniversityCleveland, Ohio; 2 Department of Genome Sciences, University of WashingtonSeattle, Washington; 3 National Hospital Organization, Murayama Medical Center, Clinical Research CenterTokyo, Japan; 4 Orthopaedic Research Laboratories, Departments of Orthopaedic Surgery and Howard Hughes Medical Institute, Children's Hospital Boston, and Department of Genetics, Harvard Medical SchoolBoston, Massachusetts

**Keywords:** Cyr61, cysteine rich protein 61, CTGF, connective tissue growth factor, Nov, nephroblastoma overexpressed, WISP, Wnt-1 inducible signaling pathway protein

## Abstract

The six mammalian CCN genes (*Cyr61*, *CTGF*, *Nov*, *WISP1*, *WISP2*, *WISP3*) encode a family of secreted, cysteine-rich, multimodular proteins having roles in cell proliferation, adhesion, migration, and differentiation during embryogenesis, wound healing, and angiogenesis. We used bioinformatics to identify 9 CCN genes in zebrafish (zCCNs), 6 of which have not been previously described. When compared with mammalian CCN family members, 3 were paralogs of *Cyr61*, 2 of *CTGF*, 2 of *WISP1*, 1 of *WISP2*, and 1 of *WISP3*. No paralog of *Nov* was found. In situ hybridization was performed to characterize the sites of expression of the zCCNs during early zebrafish development. zCCNs demonstrated both unique and overlapping patterns of expression, suggesting potential division of labor between orthologous genes and providing an alternate approach to gene function studies that will complement studies in mammalian models. Developmental Dynamics 239:1755–1767, 2010. © 2010 Wiley-Liss, Inc.

## INTRODUCTION

CCN proteins act on a variety of cell types and are involved in diverse biologic processes including development, wound healing, inflammation, and tumor growth. Putative cell biologic activities of CCNs include regulating the cell cycle, cell adhesion and migration, synthesis and remodeling of the extracellular matrix (ECM), and growth factor signaling (Leask and Abraham,^2003^). The CCN acronym is derived from the names of the first three identified family members: Cyr61, CTGF, and Nov (Bork,^1993^). It has been proposed that mammalian Cyr61, CTGF, Nov, WISP1, WISP2, and WISP3 be renamed CCNs 1–6, respectively (Brigstock et al.,^2003^).

Common to all CCN proteins are the presence of a signal peptide, specific cysteine-rich domains that have homology to domains found in other extracellular protein families, and a hinge region (Bork,^1993^). These domains resemble those present in insulin-like growth factor binding protein, von Willebrand factor, thrombospondin, and cysteine-knot containing proteins. CCNs are considered to be matricellular proteins (Bornstein,^1995^; Bornstein and Sage,^2002^; Lau and Lam,^2005^), which are proteins that principally reside in the pericellular space and functionally connect structural elements within the ECM to signaling pathways at the cell surface (Lau and Lam,^2005^; Yeger and Perbal,^2007^). Hinge regions within the CCNs contain protease cleavage sites that can generate fragments with altered biologic function (Yeger and Perbal,^2007^).

Genetically modified mice have provided insights into the roles of CCN proteins in mammals. Cyr61-null mice do not survive due to defects in placentation and large blood vessel formation (Mo et al.,^2002^). CTGF-null mice have a neonatal lethal skeletal dysplasia (Ivkovic et al.,^2003^) involving endochondral and intramembranous bone growth (Kawaki et al.,^2008^). Nov-mutant mice are viable and fertile, but develop cataracts and exhibit skeletal muscle and cardiac problems, including cardiomyopathy and muscle atrophy (Heath et al.,^2008^). However, the mutant allele in these mice produces a truncated Nov protein, making it difficult to determine if the phenotype is due to a loss-of-function or a dominant-negative effect. WISP1 and WISP2 mutant mice have not yet been reported. WISP3-null mice have no detectable phenotype (Kutz et al.,^2005^), which is surprising because loss-of-function mutations in human WISP3 cause the autosomal recessive, childhood-onset, skeletal disease, progressive pseudorheumatoid dysplasia (Hurvitz et al.,^1999^). Mice have also been generated in which CTGF, Nov, or WISP3 overexpression was driven by tissue-specific or ubiquitous promoters. Transgenic mice overexpressing Nov (Rydziel et al.,^2007^) and CTGF (Smerdel-Ramoya et al.,^2008^) under the control of the human osteocalcin promoter exhibited impaired osteoblastic function with decreased bone density and trabecular volume. This effect may be due to the overexpressed proteins antagonizing bone morphogenetic protein, Wnt, and/or insulin growth factor signaling (Smerdel-Ramoya et al.,^2008^). Transgenic mice overexpressing CTGF under the control of mouse type XI collagen promoter also showed decreased bone density as well as dwarfism (Nakanishi et al.,^2001^). Mice overexpressing WISP3 under the control of the type II collagen promoter/enhancer or ubiquitous promoters had no obvious phenotype (Kutz et al.,^2005^; Nakamura et al.,^2009^).

The zebrafish is an excellent organism for evaluating the effects of loss-of-protein function and gain-of-protein function on signaling pathways and developmental processes (Dooley and Zon,^2000^). Zebrafish are small and easy to culture, which allows large numbers of vertebrates to be maintained in a small space. Each female can lay hundreds of eggs per week, which permits large numbers to be processed. Development in zebrafish is similar to that in higher vertebrates, including human, and thus bridges the gap between invertebrate and mammalian models. Embryos develop externally and rapidly, with all the major organ systems beginning to form within 24 hours postfertilization (hpf). Embryos are translucent, which makes them easy to observe and manipulate, plus they can be visualized in vivo and investigated in real time. Because organogenesis in zebrafish resembles that which occurs in mammals, but at a more rapid rate, the effect of gene knockdown or overexpression can be evaluated in days rather than weeks. Many zebrafish mutant phenotypes resemble human disorders enabling new insights into disease processes (Dooley and Zon,^2000^; Baldessari and Mione,^2008^).

A high degree of conservation exists between zebrafish and human genomes. However, zebrafish, which are teleosts, often possess orthologous pairs of genes that are found as single copies in mammals. This is the result of tetraploidization, which occurred some time before the zebrafish and pufferfish divergence. Duplicated zebrafish genes are usually located on different chromosomes, and duplications can lead to one, or both, zebrafish orthologs having more restricted expression patterns than their mammalian counterparts (Postlethwait et al.,^2004^). This can lead to a division of labor of the zebrafish orthologs, which could not occur for the sole mammalian gene. As a consequence, a loss-of-function in a CCN family member in zebrafish may have a more restricted effect and be easier to delineate than the equivalent mutation in mammals. Thus, in anticipation of using zebrafish to further explore the in vivo functions of CCN family members, we identified and cloned several new zebrafish CCN family members and determined their endogenous expression patterns during embryonic development using in situ hybridization.

## RESULTS

### Novel CCN Genes in Zebrafish

**Fig. 1:**
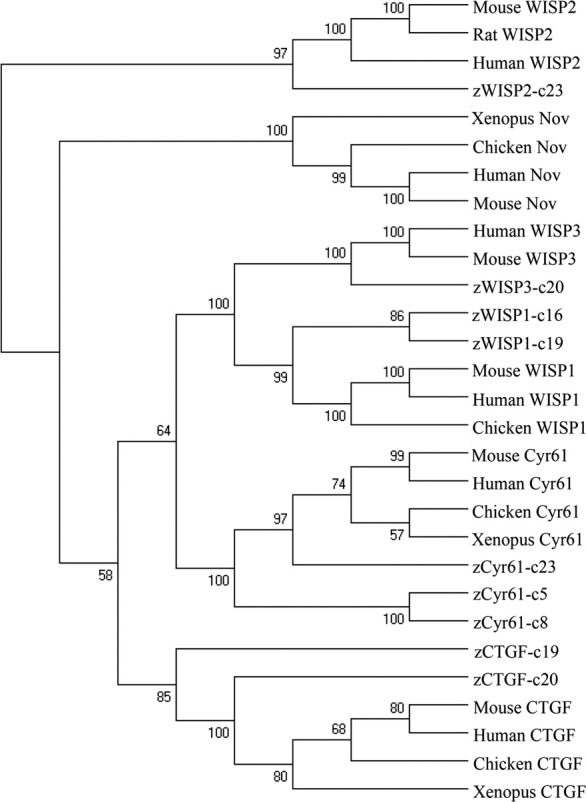
Phylogeny reconstruction of the nine members of the CCN gene family shows the position of the zebrafish genes in relation to the other mammalian members of this gene family. Numbers indicate bootstrap values.

Using the tblastn algorithm on zebrafish expressed sequence tag (EST) and nonredundant genomic DNA sequence, we identified nine genes in zebrafish that have strong homology to the mammalian CCNs (Fig. 1). Because all orthologous zebrafish CCN genes are located on different chromosomes, we have defined them by chromosome number, e.g., CTGF-c20 is the CTGF found on chromosome 20. Three of the nine genes (*CTGF-c20*, *Cyr61-c8*, and *WISP3-c20*) have been described previously (Dickmeis et al.,^2004^; Kuo et al.,^2005^; Nakamura et al.,^2007^). Among the six new genes, two had highest similarity to mammalian *Cyr61*, one to *CTGF*, two to *WISP1*, and one to *WISP2* (Fig. 1). We found no zebrafish ortholog to *Nov*. We could identify the first coding exon and the translational start site for each zCCN. Similar to their mammalian counterparts, all nine zCCNs have signal peptides.

### Expression of Most zCCNs Was Detectable by In Situ Hybridization During Embryogenesis

In situ hybridization studies were performed on embryos up to 5 days postfertilization (dpf). The stages at which specific zCCN expression was detected are summarized in Table 1.

**Table 1:** zCCN mRNA Expression During Embryonic Development Detected by In Situ Hybridization^a^

	Shield	80% epiboly	5-8 somite	24 hpf	48 hpf	50–56 hpf	70 hpf	96 hpf	120 hpf
*Cyr61-c5*	−	−	−	+	+	+	+	−	−
*Cyr61-c8*	−	−	+	−	−	−	−	−	−
*Cyr61-c23*	−	+	+	+	+	−	+	+	+
*CTGF-c19*	−	−	+	+	+	+	+	+	+
*CTGF-c20*	−	−	+	+	+	+	+	+	+
*WISP1-c16*	−	−	−	−	−	+	−	−	−
*WISP1-c19*	−	−	−	−	−	−	−	−	−
*WISP2-c23*	−	−	−	−	−	−	+	−	−
*WISP3-c20*	−	−	+	+	+	+	+	+	+

a More than 20 embryos were examined for all antisense probes, except for 50–56 hpf; at this time point only *WISP1-c16* was assayed in more than 20 embryos. Control hybridizations used sense probes for each zCCN and gave no signal.

**Fig. 2:**
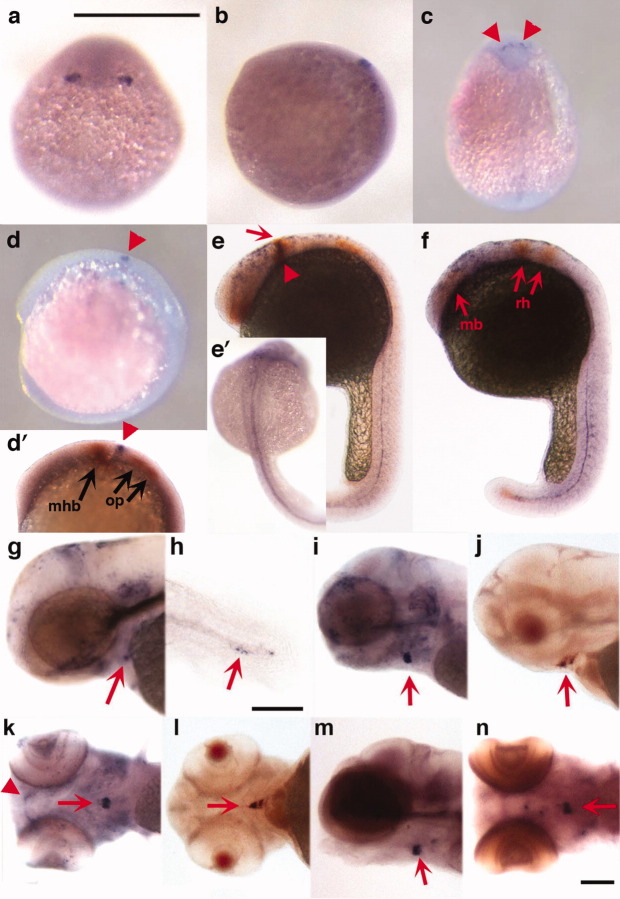
Expression pattern of *zCyr61c-23* detected by in situ hybridization. **a–n**: Photographs of zebrafish embryos staged at 80% epiboly (a, dorsal view; b, lateral view), 5–8 somites (c,d,d′), and at 24 (e,e′,f), 48 (g,h), 96 (i–l), and 120 (m,n) hours postfertilization (hpf). Expression is observed in the lateral edges of the neural plate (a,b), the dorsal brain (c,d,d′,e, arrowheads), along the midline (e′,f), somites (f), pharyngeal cartilages (k, arrowhead), thyroid region (g,i–n, arrows) and in the developing hypurals (h, arrow). Double labeling of *zCyr61c-23* (arrowhead) with *pax2a* (arrows) at five to eight somites and 24 hpf are shown in panel d′ and e, respectively. At the five to eight somite stage *zCyr61-c23* is found between the midbrain–hindbrain boundary (mhb) and the otic placode (op, d′). By 24 hpf, *zCyr61-c23* colocalizes with *pax2a* at the mhb (e, arrowhead) but is also seen extending from the anterior midbrain (mb) to the rhombomeres (rh, e, f) as evidenced by double labeling with *epha4a* (f, arrows). Double labeling of *zCyr61-c23* with *thyroglobulin* shows colocalization in the thyroid region (j, l, arrows). Scale bars = 500 μm except h, where bar = 100 μm.

Figure 2 depicts the expression pattern of *zCyr61-c23*. Embryos at 80% epiboly expressed *zCyr61-c23* at the lateral edges of the neural plate (Fig. 2a,b). At the five- to eight-somite stage, it was found in a punctate pattern across the midline (Fig. 2c,d) between the midbrain–hindbrain boundary and the otic placodes as evidenced by double in situ with *pax2a* (Fig. 2d′), an otic placode, otic vesicle, and midbrain–hindbrain marker in zebrafish (Krauss et al.,^1991^; Hans et al.,^2004^; Nechiporuk et al.,^2007^). In addition, double in situ at this stage with *epha4a*, an Eph-related receptor tyrosine kinase with spatially restricted expression in the developing fish brain (Xu et al.,^1994^), shows *zCyr61-c23* between the anterior midbrain and rhombomere 3 (data not shown). By 24 hpf *zCyr61-c23* localizes with *pax2a* specifically at the midbrain–hindbrain boundary (Fig. 2e) and also appears in a diffuse distribution extending from the anterior midbrain to the rhombomeres as evidenced by double in situ with *epha4a* (Fig. 2f). At this time point, it was also seen in the somites (Fig. 2f) and along the notochord (Fig. 2e,e′,f), and at 48 hpf in the ventral craniofacial region (Fig. 2g), where its expression persisted to 120 hpf (Fig. 2i–n). *zCyr61-c23* expression was also observed in the vicinity of the thyroid follicle (Fig. 2i–n), as determined by double in situ hybridization using a thyroglobulin probe (Fig. 2j,l). *zCyr61-c23* was also observed in the developing hypural bones (Fig. 2h).

**Fig. 3:**
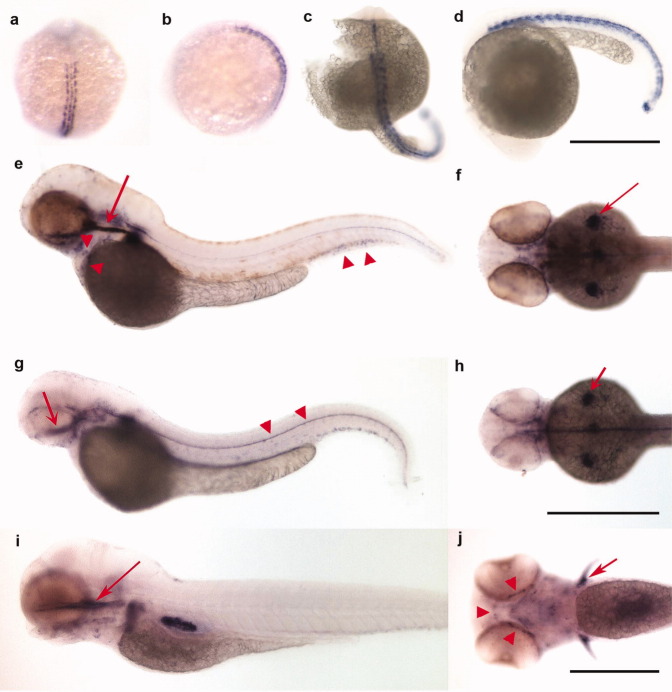
Expression pattern of *zCTGF-c20* detected by in situ hybridization. **a–j**: Photographs of zebrafish embryos staged at 5- to 8-somites (a,b), and at 24 (c,d), 48 (e,f), 70 (g,h), and 96 (i,j) hpf. Expression is observed in adaxial cells of developing somites and in the floor plate at the 5–8 somite stage (a, dorsal view; b, lateral view) and at 24 hpf (c,d). Later during development, expression is seen along the notochord (e–h, arrowhead in g), heart and axial vasculature (e, arrowheads), ethmoid plate (e,g,i, arrows), pectoral fin buds (f,h, arrows), pectoral fins and developing mandibular arch (j, arrow and arrowheads, respectively). Scale bars = 500 μm for all figures except 96 hpf, where bar = 200 μm.

Figure 3 depicts the expression pattern of *zCTGF-c20*. As had previously been reported for this zCCN (Dickmeis et al.,^2004^), it was expressed along the length of the midline in the adaxial cells of the developing somites and the floor plate at the five- to eight-somite stage (Fig. 3a,b) and at 24 hpf (Fig. 3c,d). In addition, we observed expression beginning at 48 hpf in the developing ethmoid, pectoral fin buds (Fig. 3e–j), heart, and axial vasculature (Fig. 3e,g), and along the notochord (Fig. 3e,g). At 96 hpf, it was found in the mandibular and the hyoid arches (Fig. 3i,j), which are part of the pharyngeal arches.

**Fig. 4:**
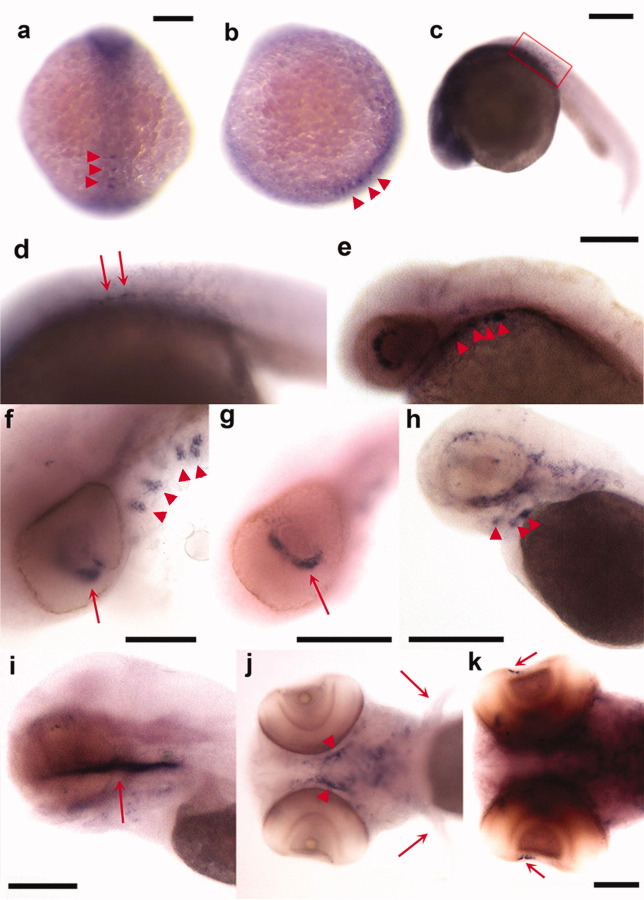
Expression pattern of *zCTGF-c19* detected by in situ hybridization. **a–k**: Photographs of zebrafish embryos staged at 5- to 8-somites (a,b), and at 24 (c,d), 48 (e–g), 70 (h), 96 (i,j), and 120 (k) hours postfertilization (hpf). Expression is observed in the neural plate and the posterior region of embryo at the midline (arrowheads in a, dorsal view, b, lateral view,), and in the posterior notochord (arrows in d, [box in c is enlarged in d]), eye (e–g,k, arrows), pharyngeal cartilages (e,f,h,j, arrowheads), and ethmoid plate (i, arrow). In contrast to *zCTGF-c20*, no *zCTGF-c19* expression is detected in the pectoral fins (j, arrow). Scale bars = 200 μm in a–c, 100 μm in f,i–k, 250 μm in e, 500 μm in g,h.

Figure 4 depicts the expression pattern of *zCTGF-c19*, which has several similarities to that of *zCTGF-c20*, as well as a few notable differences. Both are present at the five- to eight-somite stage; however, while *zCTGF-c20* showed strong expression in the somites and floor plate, *zCTGF-c19* was found in a rather diffuse distribution throughout the neural plate with the exception of a discrete punctate pattern in the posterior midline region (Fig. 4a,b). *zCTGF-c19* was expressed along the posterior notochord by 24 hpf (Fig. 4c,d), the developing pharyngeal arches beginning at 48 hpf (Fig. 4e,f,h,j), and the ethmoid plate by 96 hpf (Fig. 4i). However, unlike *zCTGF-c20*, it was not expressed in the pectoral fin buds. Unique to *zCTGF-c19* was its expression in the retina at 48 hpf (Fig. 4f,g) and in the developing lens at 120 hpf (Fig. 4k).

**Fig. 5:**
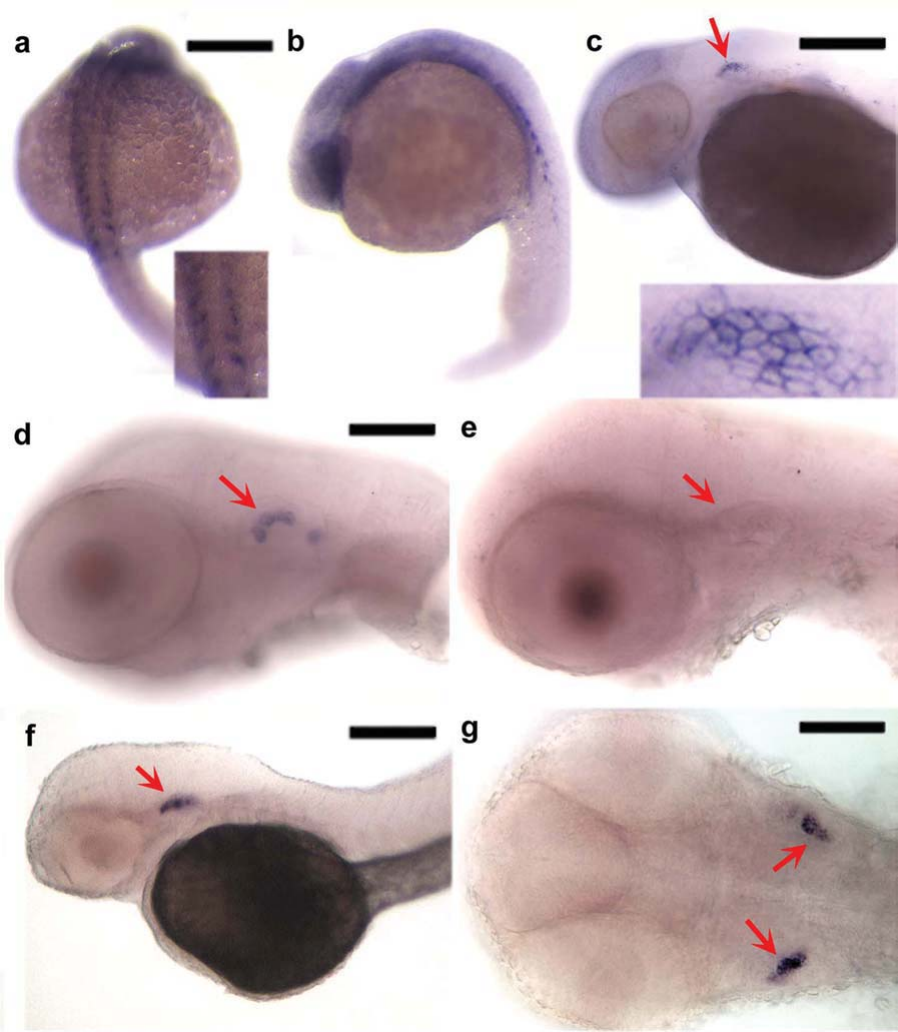
Expression pattern of *zCyr61-c5* detected by in situ hybridization. **a–g**: Photographs of zebrafish embryos staged at 24 (a,b), 48 (c), 55 (d,e), and 70 (f,g) hours postfertilization. Expression is observed in the somites (a,b) and otic vesicle (c,d,f,g, arrows). A sense probe (e), used as a negative control, demonstrates the specificity of *zCyr61-c5* expression in the otic vesicle. Inserts in a and c show higher magnification views of somites (a) and otic vesicle (c). Scale bars = 250 μm in a,b, 200 μm in c,f, and 100 μm in d,e,g.

Figure 5 depicts the expression of *zCyr61-c5*, which was first observed in the somites of 24 hpf embryos (Fig. 5a,b), and later became restricted to the epithelial layer on the roof of the otic vesicle by 48 hpf (Fig. 5c) where its expression persisted beyond 70 hpf (Fig. 5d,f,g). Although both *zCyr61-c5* and *pax2a* were found in the otic vesicle, *zCyr61-c5* was dorsal to *pax2a* (data not shown).

**Fig. 6:**
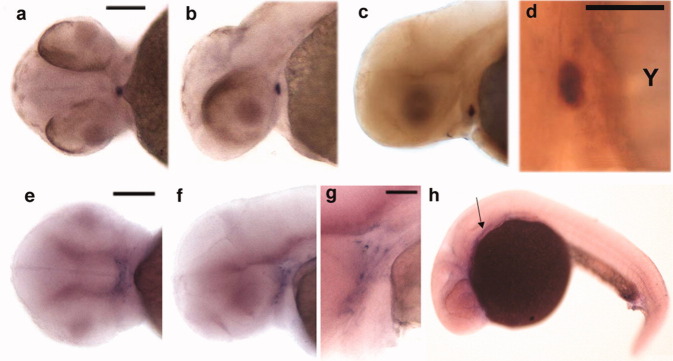
Expression patterns of *zWisp1-c16*, *zWISP2-c23*, and *zCyr61-c8* detected by in situ hybridization. **a–d**: Photographs of 52 hours postfertilization (hpf) embryos demonstrating colocalization of *zWisp1-c16* and *thyroglobulin*. *zWisp1-c16* expression is indicated in blue (a–d) and *thyroglobulin* expression in red (c,d). Y, yolk. **e–g**: Photographs of zebrafish embryos staged at 70 hpf demonstrating *zWISP2-c23* expression in the pharyngeal arches; g is at higher magnification and with a deeper focal view of the arches seen in f. **h**: Photograph of a 24 hpf embryo demonstrating *zCyr61-c8* expression in prechordal plate (arrow). Scale bars = 100 μm in a–c and g, 50 μm in d –f.

*zWISP1-c16*, *zWISP2-c23*, and *zCyr61-c8* had more restricted patterns of expression during zebrafish embryogenesis (Fig. 6). *zWISP1-c16* expression was only detected in the thyroid follicle between 50 and 56 hpf (Fig. 6a–d), based upon double in situ hybridization with *thyroglobulin* (Fig. 6d). *zWISP2-c23* expression was only found in the pharyngeal arches at 70 hpf (Fig. 6e–g). *zCyr61-c8* expression was only observed at 24 hpf in the prechordal plate (Fig. 6h).

We were unable to detect *zWISP1-c19* expression by in situ hybridization during early zebrafish development.

## DISCUSSION

We identified nine members of the CCN gene family in zebrafish, six of which are novel. As expected, due to tetraploidization in this teleost fish, the mammalian CCNs *Cyr61*, *CTGF*, and *WISP1* each have more than one ortholog in zebrafish. Gene duplication can enable a duplicated gene to acquire a different pattern of expression, a new function, or become a pseudogene (Force et al.,^1999^; Postlethwait et al.,^2004^). Each of the nine identified zCCNs likely represents a functional gene rather than a pseudogene. All have full-length open reading frames that encode proteins with signal peptides and conserved cysteine-rich domains characteristic of mammalian CCNs. Furthermore, in situ hybridization detected expression for eight of the nine zCCNs during early embryogenesis (Figs. 2–6 and Nakamura et al.,^2007^). Finding single zebrafish orthologs for the CCNs *WISP2* and *WISP3* was not surprising, since Woods and colleagues (2005) estimated that 75% of the genes that were duplicated in zebrafish have subsequently been lost.

In the developing mouse embryo, *Cyr61* was expressed in placenta, blood vessels, heart (O'Brien and Lau,^1992^), dorsal brain, floor plate, somites, notochord (Latinkic et al.,^2001^), and in condensing mesenchyme that subsequently forms skeletal elements (O'Brien and Lau,^1992^). In *Xenopus*, *Cyr61* expression was reported in somites, branchial arches, and along the notochord (Latinkic et al.,^2003^). We found that *zCyr61-c23* was expressed at several sites that have also been reported in mice and/or frogs, for example, the dorsal brain, somites, notochord, and pharyngeal arches. Furthermore, the expression we detected in the vicinity of the thyroid gland may represent expression in the ventral aorta as these two structures develop in close proximity (Alt et al.,2006a,b). Interestingly, we did not observe *zCyr61-c23* expression in otic vesicles. However, we did detect *zCyr61-c5* expression in these structures, indicating division of function may have occurred amongst the zebrafish paralogs. We do not yet know if the *zCyr61-c5* expression in otic vesicles represents a novel site of expression, or whether similar expression will be revealed by focused analyses in developing mice. The restricted expression of *zCyr61-c8* to the prechordal mesenchyme further suggests partitioning of function for the zebrafish *Cyr61* paralogs.

*CTGF* expression during mouse embryogenesis has been reported in the heart and endothelial linings of major arterial blood vessels, brain, and neural tube (Friedrichsen et al.,^2003^). These sites of expression, together with reports that CTGF promotes angiogenesis, suggest a role of CTGF as a potent angiogenic factor (Shimo et al.,^1998^,^1999^,^2001^; Brigstock,^2002^; Yang et al.,^2005^; Chintalapudi et al.,^2008^). *CTGF* expression during mouse development has also been reported in the perichondrium and chondrocytes of Meckel's cartilage of the mandibular arch (Oka et al.,^2007^). In addition, *CTGF* was found in hypertrophic chondrocytes (Nakanishi et al.,^1997^; Ivkovic et al.,^2003^) destined to form calcified tissues (Yamaai et al.,^2005^), which suggests a role for *CTGF* during terminal chondrocyte differentiation and endochondral ossification (Smerdel-Ramoya et al.,^2008^).

Our in situ findings of *zCTGF* are consistent with these interpretations. The two zebrafish orthologs *zCTGF-c19* and *zCTGF-c20* had similar patterns of expression, but they did not entirely overlap. Both were expressed in the somites and the notochord, although the expression of *zCTGF-c19* principally occurred in the posterior regions of the embryo. Localization of *zCTGF* in the notochord is consistent with other studies implicating CCN2 in notochord development (Ivkovic et al.,^2003^; Dickmeis et al.,^2004^; Chiou et al.,^2006^). Both *zCTGF-c19* and *zCTGF-c20* are found in the pharyngeal arches of the zebrafish, however only *zCTGF-c20* is found in the mandibular arch. These locations are consistent with the role of CCN2 in chondrogenesis and bone formation (Ivkovic et al.,^2003^; Dickmeis et al.,^2004^; Chiou et al.,^2006^). In zebrafish *zCTGF-c20*, but not *zCTGF-c19*, was expressed in the floor plate of the neural tube, the heart, and the developing axial vasculature. Conversely, *zCTGF-c19*, but not *zCTGF-c20*, was expressed in the developing eye. Chiou and colleagues (2006) have linked CCN2 to nervous system development in zebrafish as CCN2-morphant embryos lack eyes and have a reduced cerebrum. Whereas these results were not observed in CCN2 knockout mice (Ivkovic et al.,^2003^), CTGF did show limited expression during mouse embryogenesis to vessels lining the outer retinal surface and the retrolenticular vessels (Friedrichsen et al.,^2003^). Ocular expression of human CTGF has been described in patients with diseases of the eye (Hinton et al.,^2004^), where it may contribute to ocular neovascularization; this role, however, has been questioned (Kuiper et al.,^2007^).

In mice, *Nov* is expressed during mid-to-late stage embryogenesis in the paraxial mesoderm, which gives rise, among other tissues, to the pharyngeal arches. It is also found in chondrocytes in the growth plate (Yu et al.,^2003^) and in cranial structures derived from the neural crest (Natarajan et al.,^2000^). In chick, *Nov* is also expressed by chondrocytes (Planque et al.,^2005^) as well as by cells within the notochord and floor plate (Katsube et al.,^2001^). In humans, a major site of *Nov* expression during the first trimester appears to be the central nervous system (Kocialkowski et al.,^2001^) as both the floor plate and ventral horns of the spinal cord, spinal nerves, and dorsal root ganglia exhibit strong expression. Interestingly, we could not identify a *Nov* ortholog in zebrafish. This may be because *Nov* has been lost during zebrafish evolution, or because the zebrafish genome has yet to be sequenced in its entirety.

Mammalian *WISP1* expression has been reported in osteoblasts and osteoblastic progenitor cells of the perichondral mesenchyme (French et al.,^2004^). We could not assess whether either *zWISP1* or any other zCCNs were expressed in bone forming cells, because ossification of the zebrafish skeleton does not begin until 5 dpf (Yelick and Schilling,^2002^). We only detected *zWISP1-c16* expression in the developing thyroid gland at 50 to 56 hpf, and found no evidence for *zWISP1-c19* expression during early development.

We were only able to detect *zWISP2-c23* expression in the pharyngeal arches of developing zebrafish embryos at 70 hpf by in situ hybridization. Of interest, the cysteine-knot containing carboxy-terminal domain of WISP2 is present in zebrafish, but is missing in chicken, mouse, and human. We do not know whether this will create functional differences between the zebrafish and mammalian orthologs. However, it is likely that *zWISP2-c23* is the ortholog of the mammalian genes because it lies adjacent to the gene encoding adenosine deaminase (ADA), as does *WISP2* in humans and mice.

To date, sites of expression of mammalian *WISP3* have not been convincingly identified by in situ hybridization. Reverse transcriptase-polymerase chain reaction (RT-PCR) indicates the *WISP3* mRNA is expressed in developing kidney and in cartilage (Kutz et al.,^2005^). In developing zebrafish, *zWISP3-c20* expression was observed in the midline brain and in the otic vesicles (Nakamura et al.,^2007^).

Several zCCNs were expressed in developing cartilages. Cranial neural crest cells give rise to much of the skull and the entire pharyngeal skeleton (Knight and Schilling,^2006^). *zCTGF-c19* and *zCTGF-c20* expression was seen in the trabeculae of the ethmoid plate and *zCTGF-c20* and *zCyr61-c23* were expressed in the mandibular arch. Both structures derive from the Otx+ stream of cranial neural crest cells (Knight and Schilling,^2006^). *zCTGF-c19*, *zCTGF-c20*, *zCyr61-c23*, and *zWISP2-c23* were all expressed in the pharyngeal arch cartilages, which derive from the Hox+ stream of cranial neural crest cells (Knight and Schilling,^2006^). Surprisingly, no zCCN family member was expressed in the developing cartilage of the dorsal neurocranium, which is formed from mesodermal and cranial neural crest, and only *zCyr61-c23* expression was detected in the developing hypurals.

Having identified six new zebrafish CCN genes and determined their patterns of expression during early embryonic development, it should now be possible to explore their contributions to patterning and organogenesis using loss-of-function, graduated knock-down (e.g., morpholino mediated mRNA degradation or translation inhibition) and gain-of-function (e.g., capped mRNA injection into fertilized oocytes) approaches. These studies may complement and add new insights to those provided by the existing knockout models of the mouse CCNs. For the Cyr61 and CTGF knockout mice, their early lethality precludes studies exploring other postnatal functions of these genes. Similarly, the severe phenotypes in these mutant animals make it challenging to distinguish a direct effect of gene mutation from a secondary effect (e.g., placental insufficiency). While these limitations can be overcome using conditional knockout strategies, they may also be overcome using the zebrafish, because zCCN function may be further subdivided amongst the duplicated paralogs. For example, the unique expression pattern of *zWISP1-c16* in the developing thyroid should permit the role of this protein during thyroid development to be determined using morpholino-depletion, without affecting processes that require *zWISP1-c16* at later stages of development. Conversely, large scale tilling screens of mutagenized zebrafish will likely lead to the discovery of fish segregating loss-of-function and other classes of zCCN mutation, providing new means for delineating the in vivo functions of CCN family members during development and homeostasis.

## EXPERIMENTAL PROCEDURES

### Zebrafish Maintenance

Oregon AB and SH wild-type zebrafish (Scientific Hatcheries) were raised at 28.5°C as described in Westerfield (1994). Embryos younger than 24 hpf were staged according to Kimmel et al. (1995); older embryos were staged by hpf. To suppress pigmentation, embryos were raised in zebrafish aquatic system water containing 1-phenyl-2-thiourea (0.003%) (Sigma). Zebrafish care was in accordance with the IACUC guidelines of Case Western Reserve University and Children's Hospital Boston.

### Identification of CCN Family Genes in Zebrafish

We used the tblastn algorithm of the BLAST program (http://blast.ncbi.nlm.gov/Blast) at the National Center for Biotechnology Information to identify near matches in the zebrafish translated nucleotide database (both EST and nonredundant sequences) to each human CCN protein. Amino acid sequence identity/similarity, coupled with preservation of cysteine residue number, spacing, and cysteine-rich domain architecture was used when calling a zebrafish gene a zCCN and an ortholog of a specific mammalian CCN. Furthermore, we created phylogenetic trees by aligning zebrafish protein sequences to human, mouse, rat, chicken, and/or frog CCN sequences (Do et al.,^2005^). Trees were created using a maximum likelihood analysis and reiterated 100 times to obtain parsimony bootstrap values using RAxML (Stamatakis et al.,^2008^). Because the sequences are highly divergent, we also used GBlock to mask potential misalignments (Talavera and Castresana,^2007^). After this process of masking, topologies remained consistent but there was a reduction in statistical power to infer phylogenies (results not shown).

### 5′RACE to Obtain 5′ cDNA Ends for Each zCCN

If the first coding exon of a zCCN gene was not present within the EST database, we performed 5′ RACE (rapid amplification of cDNA ends) to identify the first coding exon and the ATG translation start site. RNA was prepared from 4 dpf AB strain embryos using Trizol Reagent (Invitrogen). Gene-specific reverse-transcription primers corresponding to known sequence from a conserved cysteine-rich domain were synthesized (Table 2). The Clontech SMART RACE cDNA amplification kit was used for first-strand cDNA synthesis. The Clontech Advantage2 PCR system was used to amplify cDNAs using the provided “universal primer” and the gene-specific primer, following the recommended touch-down thermocycling parameters. PCR products were cloned into pCR2.1 vector (Invitrogen) and several clones of each gene product were sequenced. In-frame stop codons in the 5′RACE sequence, upstream of conserved cysteines, were used to identify the most likely starting methionine of each CCN protein. The software program SignalP (Bendtsen et al.,^2004^) was used to query whether the amino acid sequence downstream of the methionine residue encoded a likely signal peptide.

**Table 2:** Gene Specific Primers Used for 5′RACE of Zebrafish CCN cDNA

zCCN	Gene specific primer for 5′RACE	bp of sequence upstream of starting ATG, obtained by 5′ RACE	Is there an in-frame stop codon upstream of starting Methionine (in 5′RACE sequence)?
*zCyr61-c5*	5′ TGATACCTGGAGGACACGAGGGGGGTGCGG	162 bp	Yes
*zCyr61-c23*	5′ GGCATCCGGAGCACAGGAGCACACCGC	114 bp	No
*zCTGF-c19*	5′ AGGGCTCTCCTGCCTGTCGTGCGCACAC	84 bp	Yes
*zCTGF-c20*	5′ GGGCGGCACTTCAGGGCAGTGGCATTGTCC	217 bp	Yes
*zWISP1-c16*	5′ ACCCCATGTGGGCAAACAAGGGCGCTCTTGG	188 bp	Yes
*zWISP1-c19*	5′ GCAGACTGACACCTGGCGGGCACATGGGA	188 bp	Yes
*zWISP2-c23*	5′ GGCAGCACTGGCACCCGTCGAGAATGAGAGGAC	267 bp	Yes

### Riboprobe Templates

RNA was prepared from 5 dpf wild-type strain SH zebrafish larvae. cDNA was made using the Superscript II RT kit with random hexamers (Invitrogen). Forward and reverse oligonucleotide primers were designed to amplify 534- to 853-bp fragments from each zCCN cDNA (Table 3). Amplicons were cloned into pCR2.1 vector (Invitrogen). Two clones of each gene insert, in opposite orientations, were used. Several clones contained one or two mismatches with the wild-type sequence, which we considered unlikely to affect riboprobe binding.

**Table 3:** Polymerase Chain Reaction Primer Pairs Used to Generate zCCN In Situ Hybridization Probes

zCCN	Forward primer	Reverse primer	Amplicon size
*zCyr61-c5*	5′CCTACTGAACCCTGCGACCACATTAAA	5′CGAGTAGTGGAACATCCAGCAAAACTG	710 bp
*zCyr61-c8*	5′GCCTAAACACACACCACAGTACAACC	5′GAAGATCGTCCTCTCTTAGCTCTGC	534 bp
*zCyr61-c23*	5′GGTGTGTGCCAGACAGCTGAAC	5′CGGCGTCACAGACTTCTTGGT	703 bp
*zCTGF-c19*	5′GTGTTAGACGGCTGTAGCTGCTGTAA	5′GGCAATTGTGATGACAGGAGCAGGTC	853 bp
*zCTGF-c20*	5′TCACCTGGTGTAAGCCTAGTTCTGG	5′GGCATGCGCAGGTCTTGATGAAC	847 bp
*zWISP1-c16*	5′CTGTGACTACAGCGCTGACAAGC	5′GCTGACACAGCCTGAGATGGTGA	568 bp
*zWISP1-c19*	5′TCAGGGAGGCTTGCAATGAGAAAG	5′GTGAAGTTTTGAGGCTTCTCTGCTCTG	604 bp
*zWISP2-c23*	5′GTGTGACTACAGCGCCAGCTTT	5′CCTGTAGCTGGGTTGACATTTCCTTC	550 bp

### Riboprobes

Ten micrograms of plasmid template was linearized with *Bam*HI, treated with proteinase K, extracted with phenol:chloroform, then chloroform, and ethanol precipitated after NaOAc addition. The DNA pellet was washed with 70% ethanol and resuspended in DEPC-treated water to approximately 0.25 μg/μl. One microgram of linearized plasmid was used in a single digoxigenin (DIG) -labeling reaction (Roche) with T7 RNA polymerase. After treatment with DNase, probes were purified on GE ProbeQuant G-50 microcolumns (Amersham) and aliquots were examined on a formaldehyde/MOPS buffer agarose gel to confirm riboprobe size and approximate concentration. Riboprobes were diluted to 4 ng/ml in DEPC-treated water, aliquoted, and stored at −80°C.

### Localizing mRNA Expression in Zebrafish Embryos

Single- and double-label whole-mount in situ hybridizations were carried out at various time points between shield and 120 hpf as previously described (Liang et al.,^2000^). Reagents were obtained from Roche Molecular Biochemicals unless otherwise indicated. For the CCN genes, probes were labeled with UTP-DIG. Embryos and probes were incubated at 70°C in hybridization solution containing 50% formamide. DIG was detected with an alkaline phosphatase (AP)-conjugated anti-DIG antibody and visualized using 4-nitro blue tetrazolium (NBT) and 5-bromo-4-chloro-3-indolyl-phosphate (BCIP [blue precipitate]). The reactions were stopped by replacing substrate with several rinses of PBT (phosphate-buffered saline: PBS, 0.1% Tween 20) and then stored in PBS containing 0.02% sodium azide in the dark at 4°C until photographed.

For *epha4a*, *pax2a*, and *thyroglobulin* (*tg*), the probes were labeled with UTP-fluorescein, detected with the appropriate AP-conjugated antibody, and visualized using iodo-nitrotetrazolium violet [INT, Sigma (orange precipitate)] in AP buffer (100 mM Tris, 50 mM MgCl_2_, 100 mM NaCl, 0.1% Tween-20, pH 9.5) containing 10% polyvinyl alcohol (PVA, Sigma). The reaction was stopped by rinsing in AP buffer, followed by PBT, and embryos were stored in 4% paraformaldehyde (PFA). For double-labeling, embryos were hybridized simultaneously with two probes (*Cyr61-c23* and *tg*, *epha4a*, or *pax2a*, and *WISP1-c16* and *tg*) labeled with DIG and fluorescein, and the first probe was developed with NBT/BCIP as described above. To inactivate AP conjugated to the first antibody, embryos were post-fixed overnight at 4°C in 4% PFA, washed twice for 20 min with PBT, heated for 10 min at 70°C in 10 mM ethylenediaminetetraacetic acid in PBT, and dehydrated in methanol for 10 min. Following rehydration through a methanol/PBT series, the samples were washed 4×, 10 min each, in PBT, blocked for 1 hr in blocking buffer (PBT, 2% sheep serum, 0.2% BSA), and incubated overnight at 4°C with appropriate antibody diluted 1:5,000 in blocking buffer. After washing 6× for 15 min each in PBT, and 3× for 5 min each in AP buffer, the second probe was visualized with BCIP and 0.2 mg/ml INT in AP buffer with 10% PVA.

### Microscopy

Embryos were mounted on glass slides in 50%glycerol/PBS and observed with brightfield optics on either a Leica DMLB upright microscope (5×, 0.15N.A.; 10×, 0.3N.A.; 20×, 0.5N.A.) or a Leica MZ15F stereomicroscope. Images were acquired with a SPOT RT (Diagnostic Instruments, Inc.) slider color digital camera.

### Zebrafish CCN Genes With Genbank Accession Number

*zCyr61-c5*: paralog of mammalian *Cyr61* on zebrafish chromosome 5, GQ273493; *zCyr61-c8*: paralog of mammalian *Cyr61* on zebrafish chromosome 8, GQ273499; *zCyr61-c23*: paralog of mammalian *Cyr61* on zebrafish chromosome 23, NM_001001826; *zCTGF-c19*: paralog of mammalian *CTGF* on zebrafish chromosome 19, GQ920789; *zCTGF-c20*: paralog of mammalian *CTGF* on zebrafish chromosome 20, NM_001015041; *zWISP1-c16*: paralog of mammalian *WISP1* on zebrafish chromosome 16, GQ273496; *zWISP1-c19*: paralog of mammalian *WISP1* on zebrafish chromosome 19, GQ273497; *zWISP2-c23*: paralog of mammalian *WISP2* on zebrafish chromosome 23, GQ273495; *zWISP3-c20*: paralog of mammalian *WISP3* on zebrafish chromosome 20, GQ273498.
